# Functional Characterization of Rice Spotted-Leaf Mutant *HM113* Reveals an Amino Acid Substitution in a Cysteine-Rich Receptor-like Kinase

**DOI:** 10.3390/plants14223429

**Published:** 2025-11-09

**Authors:** Ringki Kuinamei Sanglou, Marie Gorette Kampire, Xia Xu, Jian-Li Wu, Junyi Gong, Xiaobo Zhang

**Affiliations:** State Key Laboratory of Rice Biology and Breeding, China National Rice Research Institute, Hangzhou 311400, China; ringki007@gmail.com (R.K.S.); kamgorette@gmail.com (M.G.K.); mailxuxia@163.com (X.X.); beishangd@163.com (J.-L.W.)

**Keywords:** rice, spotted leaf, bacterial blight, defense response, resistance, reactive oxygen species

## Abstract

The spotted-leaf mutant, characterized by spontaneous lesion formation resembling pathogen-induced hypersensitive cell death, serves as an ideal model for studying the molecular mechanisms behind rice (*Oryza sativa*) disease resistance and programmed cell death, as these plants display hypersensitive responses that mimic those triggered by pathogen infection. In this study, we generated a knockout line using CRISPR/Cas9 technology in homologous mutant *HM113*-induced calli. *LOC_Os07g30510* encodes a cysteine-rich receptor kinase with a DUF26 domain, consisting of 688 amino acids. *HM113* was localized to the cytosol and expressed in most rice tissues at various growth stages. A single nucleotide substitution from A to T was observed at the 847th base of *LOC_Os07g30510*, causing an amino acid change from serine to cysteine. Our results demonstrated that the A847T mutation was responsible for the spotted-leaf phenotype in the *HM113* mutant through gene editing technology, as new frameshift mutations were introduced upstream of the A847T site in the *HM113* gene. The mutation phenotype of *HM113* was eliminated and resistance to bacterial blight was also lost, indicating that it is a gain-of-function gene.

## 1. Introduction

Rice, also known as *Oryza sativa* L., is the most widely cultivated and ancient agricultural crop globally, consumed by rural and urban people, and the primary food source for nearly half of the global population [1]. Bacterial blight (BB) is a severe disease that affects rice crops, significantly impacting global rice production and resulting in substantial losses and damage. The estimated yield losses due to this disease range from 20% to 80%, posing a significant threat to rice production [2,3,4]. Spotted-leaf mutants are a crucial class that exhibit spontaneous cell death, resulting in necrotic lesions or spots on leaves, regardless of whether the stress is biotic or abiotic [5]. These mutants are also known as lesion-mimic mutants (LMMs) and display spontaneous lesions in the absence of pathogen attack, environmental stress, or mechanical damage. Many LMMs exhibit constitutive activation of defense pathways, resulting in enhanced resistance to specific pathogens [6,7].

In rice, several LMMs have shown increased resistance to the blast fungus *Magnaporthe oryzae*. Several lesion-mimic mutants in rice, such as *oscul3a* and *LIL1*, have shown ROS accumulation, increased expression of disease-related genes, and significant resistance to *Magnaporthe oryzae* and *Xanthomonas oryzae* pv. *oryzae* (*Xoo*). These include the upregulation of pathogenesis-related (PR) genes, accumulation of reactive oxygen species (ROS), and higher expression of salicylic acid (SA)- and jasmonic acid (JA)-dependent signaling genes [8,9]. Likewise, disease resistance to bacterial blight caused by *Xanthomonas oryzae* pv. *oryzae* (*Xoo*) is higher in LMMs than in wild-type cultivars such as IR64, due to constitutive expression of defense-related genes and an increase in basal immune responses [6]. The symptoms are similar to those caused by pathogens or an infection-induced hypersensitive response (HR), hence the term “spotted-leaf mutants” [10]. Plants have developed a host defense mechanism against these pathogens; one of the most common is the hypersensitive response (HR), a crucial innate immune response that involves the rapid induction of localized cell death during an incompatible interaction between a plant and a pathogen [11]. The hypersensitive response is characterized by a burst of reactive oxygen species (ROS), the expression of pathogenesis-related (PR) genes, the accumulation of phytoalexins or antimicrobial compounds, and alterations in the cell wall, all of which contribute to the suppression of pathogen development [12]. Programmed cell death (PCD) is followed by disease-like spots, which trigger the plant’s defensive response, enhance the expression of defensive genes, and promote plant tolerance to diseases such as rice blast and bacterial blight. Similarly, lesion mimics mutants provide natural resources for understanding the processes of plant-programmed cell death and defensive responses [13].

In recent years, many rice LLM genes have been discovered and isolated. Over 80 spotted-leaf mutants have been identified, with most being recessive and some regulated by dominant or semi-dominant genes. *Spl 40* [14], *HM47* [15], and *HM143* [16] are one gene controlled by a single recessive nuclear gene. *Spl26*, the spotted-leaf trait, is governed by a dominant nuclear gene on the short arm of chromosome 7 [17]. The gene *Spl24* is known to be semi-dominant [18].

They also encode various proteins with diverse roles and functional categories related to rice defense responses and HR cell death signaling mechanisms. These proteins, encoded by spotted-leaf genes in rice, include membrane-associated proteins [19], splicing factor 3 subunit 3 [20], U-box domain-containing proteins [21], and splicing factor 3b subunit 3 (SF3B3), a protein found in *Spl5* that might be involved in the early splicing of RNAs, which regulates defense mechanisms and cell death [20]. Additionally, *Spl7* encodes a heat stress transcription factor protein that confers resistance to environmental stress [22]. It was found that *Spl11*, one of the most extensively studied rice LMMs, encodes a U-box/Armadillo repeat protein with E3 ubiquitin ligase activity and resistance to various diseases. Ubiquitination may play a role in managing plant programmed cell death (PCD) and pathogen defense [23]. These findings suggest that several proteins work together to prevent HR cells from dying and to enhance disease resistance.

Many genes that produce receptor-like protein kinases (RLKs) were also cloned. The plasma membrane-localized receptor-like kinases (RLKs) play a key role in sensing and transducing external stimuli to activate downstream signaling pathways [24]. The rice gene *Xa21*, a unique disease-resistance gene, encodes receptors similar to kinases with leucine-rich repeats in the extracellular, transmembrane, and serine-threonine protein kinase domains, contributing to understanding disease resistance. This gene is also resistant to multiple races of *Xanthomonas oryzae* pv. *oryzae* (*Xoo*) [25]. The Rice *Spl36* gene regulates the defense response of rice and encodes a protein kinase with receptor-like properties. The loss-of-function mutant *Spl36* exhibits an enhanced resistance to the rice bacterial blight strain *HM73* [26]. The *Lmm24,* which regulates cell death and defense responses, encodes a receptor-like cytoplasmic kinase [5]. Another resistance gene, Pto, and the Arabidopsis receptor-like kinase 902 (AtRLK902) carry a protein kinase domain. Pto confers resistance to *Pseudomonas syringae* pv tomato (Pst) [27], and AtRLK902 resists infection by the bacterial pathogen Pseudomonas syringae [28].

We previously identified a new dominant homozygous lethal spotted-leaf mutant, *HM113*, derived from ethyl methane sulfonate (EMS) mutagenesis of IR64. The mutant *HM113* was fine-mapped to the long arm of chromosome 7. Three weeks after sowing, the mutant *HM113* developed brown lesions on its leaves, showed programmed cell death, and exhibited increased resistance to multiple bacterial blight pathogen races [29]. Here, we demonstrated that the mutation caused the spotted-leaf phenotype in *HM113* and that *LOC_Os07g30510* is the target gene of the dominant mutant, confirmed through gene editing technology. Specifically, editing the upstream mutation site of the *HM113* gene resulted in frameshift mutations; the mutation phenotype disappeared, along with resistance to bacterial blight. This indicates that it is a gain-of-function gene.

## 2. Results

### 2.1. HM113 Was Cloned, and It Encodes a Cysteine-Rich Receptor Kinase That Has a DUF26 Domain

**Figure 1:**
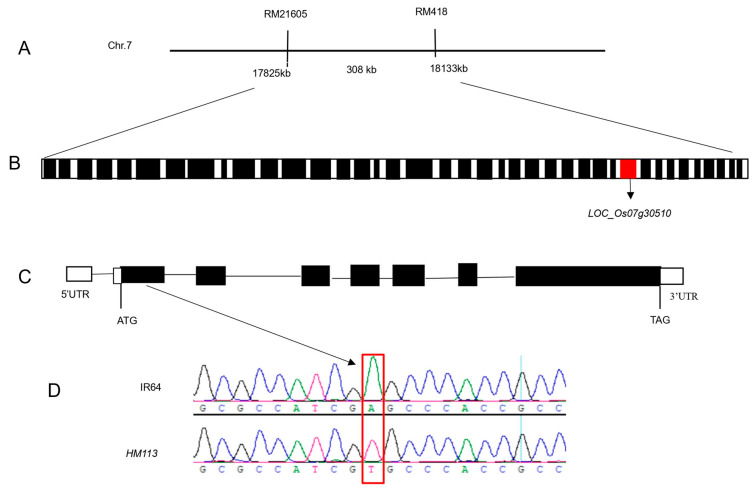
Map-based cloning of *HM113.* (**A**) The gene *HM113* is positioned on chromosome 7, specifically between the markers RM21605 and RM418. (**B**) In the 308 kb area, 39 ORFs were identified. The red box indicates *LOC_Os07g30510*, while the other black boxes represent different open reading frames (ORFs). (**C**) *LOC_Os07g30510* gene structure: white boxes are 5’UTR and 3’UTR, black boxes are exons, and lines are introns. (**D**) A to T point mutation sequence analysis in WT IR64 and *HM113* in the 1st exon.

The novel single dominant nuclear gene *SPLHM113* was mapped to the long arm of chromosome 7 by [29]. The previous study obtained 698 F2 individual plants from *HM113*/Moroberekan and *HM113*/CPSLO17 F2 populations for fine mapping. The gene *SPLHM113* was located between RM21605 and RM418, with a physical distance of about 308 kb (Figure 1A). Additionally, the mapped region contained 39 potential open reading frames (ORFs), as shown in the Rice genome annotation project database (http://rice.plantbiology.msu.edu/ (accessed on 23 June 2024)), seen in (Figure 1B). Among these, *LOC_Os07g30510,* highlighted in red in (Figure 1B), was identified as a candidate gene. To identify potential SNPs or indels linked to the dominant mutant phenotype, we used NGS technology. The reference genome was the indica rice MH63, and we resequenced the genomes of *HM113* and three other IR64 background lines. This analysis produced four VCF files. Using Excel’s “vlookup” function, we compared the SNPs within the 308 kb candidate region. A SNP may be the mutation we are searching for if it is unique to the *HM113* mutant. The same method was applied to compare indel VCF files; however, no *HM113*-specific indels were detected. From this analysis, we identified 19 SNPs specific to *HM113*; of these, 7 SNPs were located in exonic regions, and 5 of these were nonsynonymous, resulting in amino acid substitutions (supplemental data Table S2). For confirmation, we used Sanger sequencing after designing primers to amplify each of the seven exonic SNP-containing regions by PCR. This confirmed that only a single nonsynonymous SNP was consistently associated with the mutant phenotype. As shown in (Figure 1C), the mutation was confirmed in the first exon of *LOC_Os07g30510* through PCR amplification and sequencing. The nucleotide mutation changed from A to T at position 847, resulting in an amino acid change from serine to cysteine (Figure 1D). The gene consists of 7 exons and 6 introns, with a total length of 4054 nucleotides. It is predicted that *LOC_Os07g30510* encodes a cysteine-rich receptor kinase containing a domain of unknown function 26. As a result, *LOC_Os07g30510* was identified as a candidate gene responsible for the mutant phenotype.

### 2.2. HM113 Is Widespread Expressed and HM113 Localizes to the Cytosol

**Figure 2:**
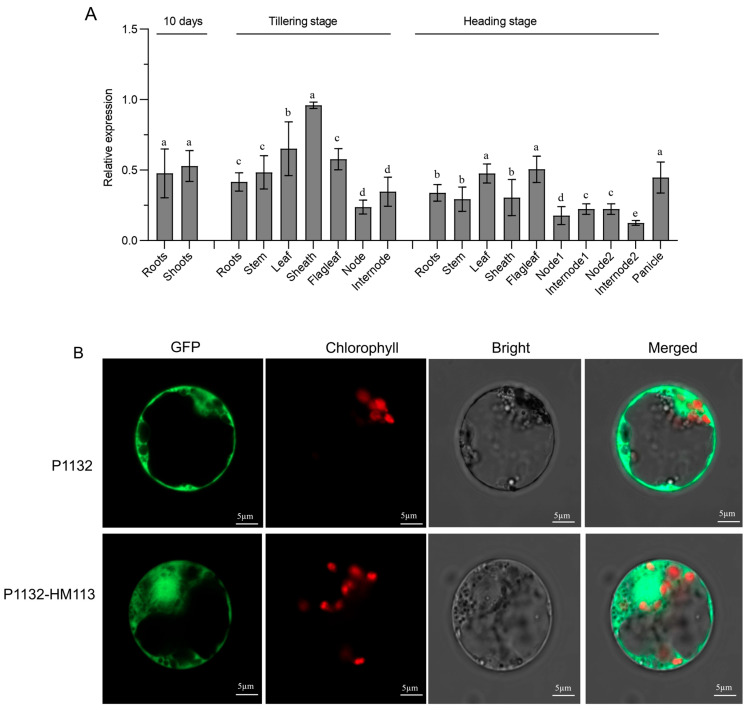
Relative Expression Analysis of *HM113* in Wild-Type IR64 and Subcellular Localization of HM113 in Rice Protoplasts. (**A**) The quantitative relative expression level of the *HM113* gene was determined in various tissues of wild-type IR64 rice at both the 10-day, tillering stage and the heading stage. Different letters indicate significant differences at *p* < 0.05 by Duncan’s multiple test. (**B**) The top picture (P1132) represents the GFP signal in rice protoplasts alone. Bars = 5 µm. The bottom image (P1132-HM113) represents the HM113 fusion protein in rice protoplasts. Bars = 5 µm.

The quantitative reverse transcription-polymerase chain reaction (qRT-PCR) was used to measure the relative expression of *HM113* at different developmental stages of IR64. Total RNA was extracted from various tissues of the wild-type IR64 at different stages and then converted into complementary DNA (cDNA). Our results showed that HM113 was widely expressed across all examined tissues, with the highest expression levels observed in the sheath during the tillering stage (Figure 2A). These findings indicate that HM113 is a broadly expressed gene.

To determine the subcellular localization of HM113, we performed rice protoplast transformation using polyethylene glycol (PEG) as a mediator. The coding sequence of HM113 was fused with green fluorescent protein (GFP) at its N-terminal. When transiently expressed in rice protoplasts, the GFP signal was confined to the cytosol (Figure 2B). Our data clearly showed that HM113 is a protein localized in the cytosol.

### 2.3. The Phenotype Characterization of Knockout Lines in HM113

**Figure 3:**
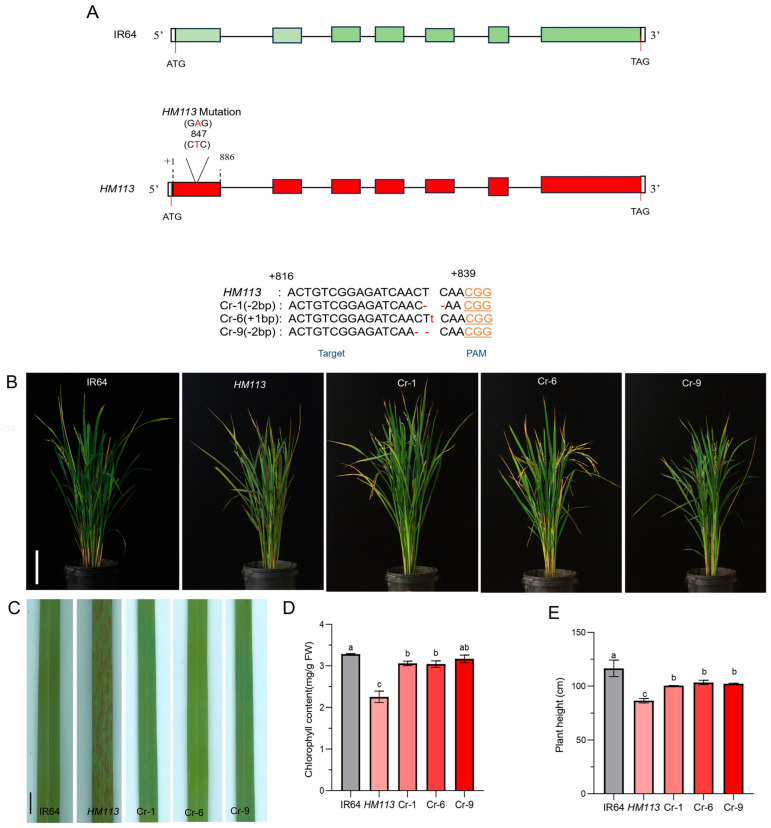

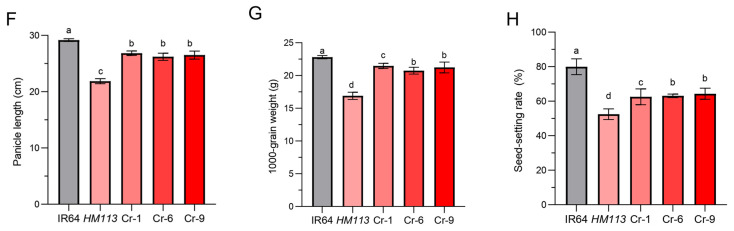
Phenotypic identification and functional validation of the *HM113* knockout strain. (**A**) CRISPR/Cas9-mediated mutation at the target site of knockout lines (Cr-1, Cr-6, and Cr-9), small red letters indicate the corresponding base insertions, dot lines indicate deletions; (**B**) Plant phenotypes of wild-type IR64, mutant *HM113*, and knockout lines Cr-1, Cr-6, and Cr-9 at the filling stage. Bar = 15 cm. (**C**) Leaf Phenotype of wild-type IR64, mutant *HM113*, and knockout lines Cr-1, Cr-6, and Cr-9 at the filling stage. Bar = 2 cm. (**D**) Chlorophyll content. (**E**) Plant height (**F**) Panicle length (**G**) 1000-grain weight (**H**) and Seed setting rate of wild-type IR64, mutant *HM113*, and knockout lines Cr-1, Cr-6, and Cr-9. Values are means ± SD, (n = 3). Different letters indicate significant differences according to One-way ANOVA and Duncan’s test (*p* < 0.05).

We used CRISPR/Cas9 to verify the function of the target gene in the *HM113* mutant background. The background was checked to ensure that the seeds are not heterozygous. The genotype was confirmed by PCR detection at the specific mutation site of *HM113*, at position 847, involving an A-to-T mutation. A total of 15 independent lines were generated through Cas-9-mediated editing. Three independent knockout lines (Cr-1, Cr-6, and Cr-9) with growth characteristics similar to those of IR64 were selected for further study. Further sequencing of these lines revealed that Cr-6 had a 1 bp insertion, while Cr-1 and Cr-9 had a 2 bp deletion at the target site, leading to a frameshift mutation (Figure 3A).

Through these phenotype observations, we found that when the gain-of-function gene *HM113* homozygous mutant was knocked out using the CRISPR/Cas9 method, the plants did not exhibit a spotted-leaf phenotype and displayed a similar phenotype to the wild type (Figure 3B,C). The total chlorophyll content of Cr-1, Cr-6, and Cr-9 was the same as that of the wild type, and their chlorophyll levels were significantly higher than in the mutant *HM113* (Figure 3D). Although the other agronomic traits of the knockout lines did not fully return to wild-type levels, they demonstrated significant improvement compared to the mutant *HM113* (Figure 3E–H). Taken together, these results indicate the efficiency of CRISPR/Cas9 and confirm that *LOC_Os07g30510* is our target locus, responsible for the lesion mimic phenotype in *HM113*.

### 2.4. Reactive Oxygen Species Accumulation in the Knockout Lines

It has been shown that, at the physiological level, ROS play a crucial signaling role in plant growth and development. However, excessive ROS can cause oxidative damage to proteins, lipids, and nucleic acids, leading to lesion formation and cell death [30]. To verify our findings, we measured physiological and biochemical indicators in the wild-type IR64, the mutant *HM113*, and the three knockout lines Cr-1, Cr-6, and Cr-9. We evaluated whether they exhibited similar plant phenotypes to those of the wild-type or the mutant.

**Figure 4:**
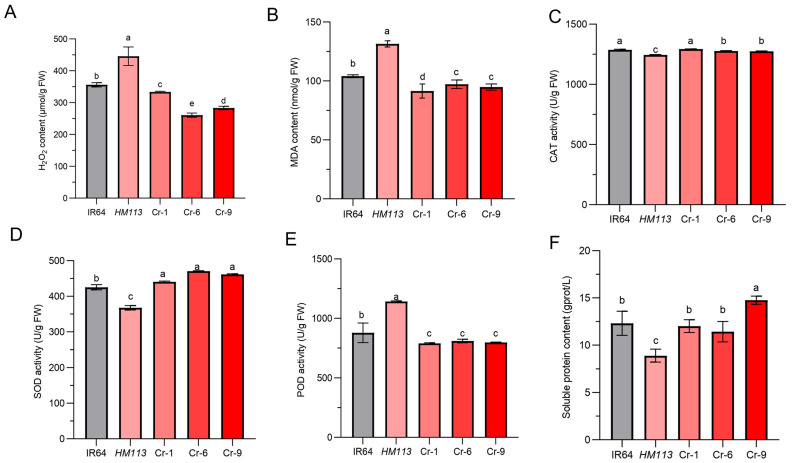
Physiological performance of transgenic lines, mutants, and wild types at the tillering stage. (**A**). H_2_O_2_ content (**B**). MDA content (**C**). CAT activity (**D**). SOD activity (**E**). POD activity (**F**). Soluble protein content. Data are means ± SD (n = 3). Different letters indicate significant differences according to Duncan’s multiple test (*p* < 0.05).

The hydrogen peroxide content (H_2_O_2_) in the wild-type IR64 and the knockout transgenic lines Cr-1, Cr-6, and Cr-9 was significantly lower compared to the mutant *HM113* (Figure 4A). This indicated increased oxidative stress. The malondialdehyde (MDA), which measures membrane lipid peroxidation, was highest in the mutant *HM113* compared to the MDA concentrations in IR64, Cr-1, Cr-6, and Cr-9, which remained stable and exhibited lower concentration than the mutant *HM113*, suggesting less membrane damage (Figure 4B).

Furthermore, we examined the reactive oxygen species scavenging enzymes, including catalase (CAT), superoxide dismutase (SOD), and peroxidase (POD), along with their respective content levels. While the activities of catalase (CAT) (Figure 4C) were significantly lower in *HM113*, the three knockout lines recovered similarly to the wild-type, indicating a limited capacity for ROS detoxification. The activity of Superoxide dismutase (SOD) was the lowest in the mutant *HM113* compared to the wild type IR64 and the knockout lines Cr-1, Cr-6, and Cr-9 (Figure 4D). At the same time, the Peroxidase (POD) level was higher in the mutant *HM113* compared to the wild type IR64 and the knockout lines Cr-1, Cr-6, and Cr-9 (Figure 4E), indicating that the gain-of-function by *HM113* is causing the disorder in the ROS scavenging system. Furthermore, the soluble protein content in the knockout lines Cr-1, Cr-6, and Cr-9 also returned to wild-type levels (Figure 4F). In conclusion, the results indicate that knocking out the mutant alleles can restore the normal functions of the reactive oxygen species scavenging system to normal levels in the knockout lines, thereby preventing the accumulation of reactive oxygen species and preventing oxidative damage to cells. This also suggests that the dominant mutation in the *LOC_Os07g30510* gene can increase ROS accumulation in cells, triggering cell damage and death, which leads to lesion formation in *HM113*.

### 2.5. Knockout Line Exhibits Resistance Comparable to Wild Type

**Figure 5:**
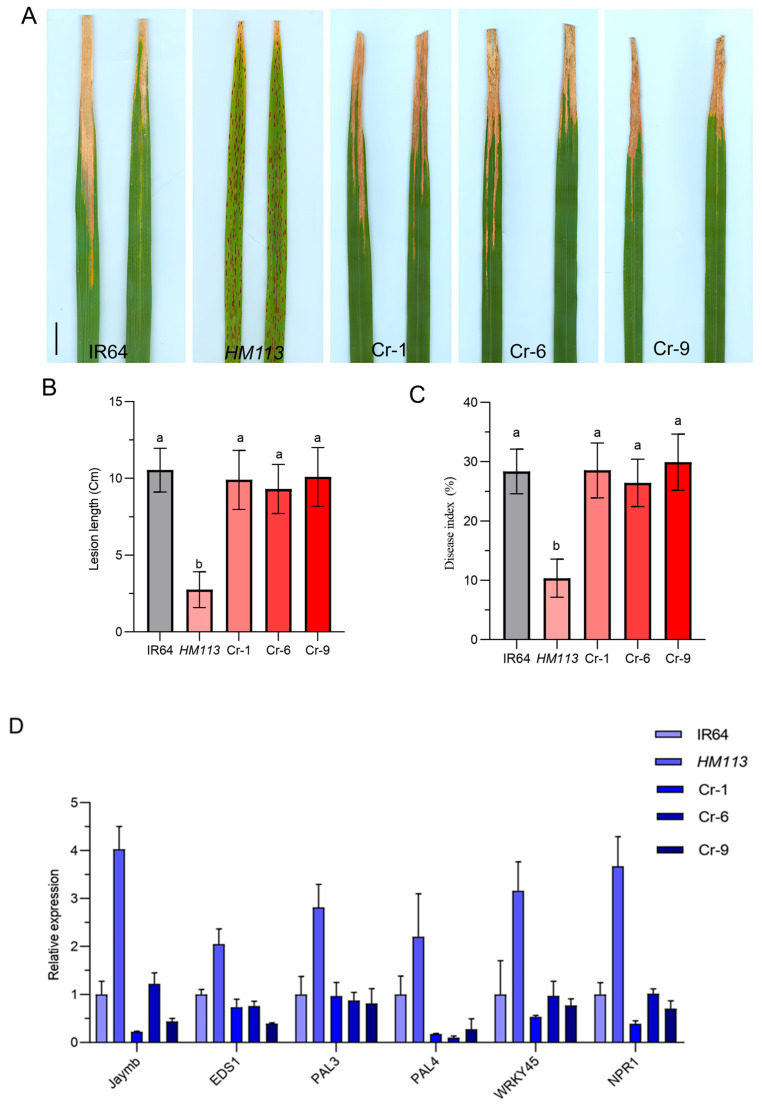
Disease evaluation and relative expression of defense response genes. (**A**) Responses of Wild-type IR64, mutant *HM113*, and knockout lines to bacterial blight race *PXO37* during the tillering stage. Bar = 5 cm; (**B**). Lesion Length; (**C**). Disease Index; Different letters indicate significant differences at *p* < 0.05 by Duncan’s multiple test. (**D**). Analysis of defense response gene expression levels in wild-type, mutant, and knockout lines, at the tillering stage. Data are means ± SD (n = 3).

Previous studies indicated that *HM113* exhibits disease resistance to multiple races, while *PX0347* demonstrates the highest level of resistance [29]. To determine that the enhanced resistance resulted from the mutation of *HM113*, the knockout lines, the mutant, and the wild types were inoculated with the bacterial blight strain *PX0347.* We found that the knockout lines Cr-1, Cr-6, and Cr-9 were all infected with the same pathogen as the wild-type. They showed significantly higher lesion length and disease index compared to the mutant *HM113,* indicating their resistance to the pathogen (Figure 5A–C). These results showed that knocking out the gene of the function mutant can reduce the resistance of plants to bacterial blight, and the mutant allele is responsible for the increased resistance to *HM113.*

To further assess the enhanced disease resistance associated with the *HM113* mutation, gene expression levels of defense response genes [29] in the IR64 wild-type, *HM113* mutant, and knockout lines were measured by qRT-PCR. According to the results of qRT-PCR, we found that the expression level of the defense response genes in the knockout lines was significantly lower compared to the mutant *HM113*, indicating that after knocking out the mutant allele, the defense response cannot be activated as effectively as in the mutant, which led to a decrease in the disease resistance of the knockout lines to the bacterial blight pathogen (Figure 5D). These findings demonstrated that the gain-of-function mutation initiates the rice defense response and that *HM113* enhances rice resistance to *Xoo*. A dominant mutation in the mutant *HM113* can promote ROS accumulation, cell death, and an immune system response.

## 3. Discussion

The spotted-leaf mutants spontaneously develop necrotic lesions in plants, which impact production and provide a unique opportunity to study programmed cell death in plant defense mechanisms. Previously, we isolated a spotted-leaf mutant from the EMS-induced mutant library of indica rice IR64 and found that the gene *LOC_Os07g30510* may control the *HM113* spotted-leaf phenotype. Brown lesions were observed on the leaves three weeks after sowing in natural conditions, spreading to the sheaths during the heading stage. The mutant also exhibited enhanced disease resistance to several races of *Xoo* strains [29]. In the present study, to understand the function of *HM113* in rice, we generated a knockout line using CRISPR/Cas9 technology in the homologous mutant *HM113*-induced calli.

Despite its poor agronomic traits, the rice spotted-leaf mutant can achieve high yields by enhancing photosynthetic efficiency, disease resistance, and response to environmental stress [30]. In this study, as seen in some of the lesion mimic mutants, *LMMs* like *Lmm28* [31], *Spl24* [18], *Spl36* [26], and *Spl26* [32] exhibited reduced agronomic characteristics, including plant height, panicle length, and grain yield, in rice due to disruptions in ROS regulation and programmed cell death, resulting in lower agronomic traits in the plants.

Similarly, the mutation significantly decreased all the agronomic characteristics studied in the mutant *HM113* compared to the wild-type IR64, impacting yield and its components. However, we found that knocking out the *HM113* mutant gene in transgenic knockout lines increased the agronomic traits, and the chlorophyll content returned to levels similar to the wild type, which was comparable to other rice lesion mimic mutants, *Spl26* [17] and *Spl42* [33]. This finding suggests that the *HM113* mutation can directly influence lesion formation and indirectly affect agronomic performance, indicating that a dominant mutation can lead to reduced yield.

The development of lesions is due to the excessive buildup of H_2_O_2,_ which is the primary factor responsible for it [5]. The degree of membrane cellular damage can be indirectly reflected by the accumulation of MDA, which is one of the most critical products of membrane lipid peroxidation [34]. Similarly, *HM113* had elevated levels of MDA and H_2_0_2_, suggesting that the accumulation of cell death may have contributed to the development of necrotic lesions. The oxidation-reduction reaction within plant cells generates reactive oxygen species (ROS). A novel lesion mimic mutant *LIL1***,** which also displayed spontaneous light-induced lesions independent of pathogen attack. These lesions were associated with reactive oxygen species ROS accumulation and programmed cell death (PCD)**,** and with upregulated expression of defense-related genes, particularly PR1, PR10, POX22.3, and POC1 [9]. However, gene mutations disrupt the plant’s internal environment in many spotted mutants, affecting the balance of ROS production [35,36,37]. The mutation in *HM113*, A847T (*LOC_Os07g30510*), disrupted the balance of reactive oxygen species (ROS). Here, we observed a significant decrease in CAT and SOD levels, while the POD level was upregulated, leading to ROS accumulation. This caused the accumulation of H_2_0_2_, which acts as a signaling molecule that activates programmed cell death (PCD) pathways in plants [5]. We further measured multiple metacaspase genes. The metacaspase gene encodes a cysteine-dependent protease that regulates cell death in plants. Our results indicated that the mutant *HM113* expressed a high level of these metacaspase genes (*OsMC2*–*OsMC7*) (Supplemental Data Figure S2), which are essential regulators of PCD in plants [38]. This observation revealed the biological connection between the mutation of *LOC_Os07g30510*, ROS, and PCD. Additionally, this further demonstrates how the mutation in *LOC_Os07g30510* leads to excessive ROS accumulation, resulting in increased metacaspase expression and the development of PCD, cell death, and this elucidates the conclusion that *LOC_Os07g30510* controls ROS signaling and PCD.

In this study, the initially observed spotted-leaf phenotype disappeared when we knocked out the gain-of-function gene *HM113*. These results suggest that a dominant mutation in *HM113* can induce cell death, resulting in the spotted-leaf phenotype. ROS signals play a crucial role in plant development; however, excessive amounts in rice *LMMs* lead to cell death and lesion formation [17]. For instance, some dominant genes, such as *Spl26*, exhibit increased ROS levels, enhanced disease resistance, and upregulated defense-related genes [17]. Similarly, *spl11* demonstrates an elevated defense response, and its mutation results in lesion formation. The protein U-box/ARM, encoded by this gene, regulates cell death [23]. Another gain-of-function gene, *Spl7*, enhances disease resistance and affects agronomic traits such as plant height and grain yield [22]. The *LIL1* mutant also displayed increased resistance to rice blast fungus (*Magnaporthe grisea*) [9]. Previous studies have shown that H_2_O_2_ is produced in response to various environmental and developmental stimuli, which are essential for regulating programmed cell death in plants [39]. In our studies, the mutant *HM113* exhibited higher levels of H_2_O_2_, resulting in severe cell death, which suggests that the toxic accumulation of H_2_O_2_ contributes to cell death in plants. However, after knocking out the mutated gene, the H_2_O_2_ levels returned to normal, and reactive oxygen species functioned similarly to those in the wild type. These findings imply that the H_2_O_2_ buildup in mutant *HM113*, or the mutation itself, may be caused by the mutant gene. The knockout line also exhibited the same level of MDA content as the wild type, which is induced by reactive oxygen species and membrane lipid peroxidation. Overall, *HM113* indicates that the dominant mutation in the *HM113* gene disrupts the intracellular reactive oxygen species scavenging system, leading to excessive H_2_O_2_ accumulation and ultimately triggering programmed cell death, resulting in spotted-leaf phenotypes [40]. Nevertheless, further research is needed to elucidate the specific molecular pathways through which the *HM113* mutation affects the reactive oxygen species scavenging system.

Studies have shown that the formation of lesions in spotted-leaf mutants often increases disease resistance [5,18,21,40]. Generally, excessive ROS buildup causes lesion formation, activating defense response genes and increasing resistance to disease pathogens [6,30]. In this study, the mutant allele *HM113* induced lesion formation, upregulated the expression of numerous defense response genes, and enhanced plant resistance to bacterial blight pathogens. Salicylic acid (SA) and jasmonic acid (JA) are crucial regulators of plant defense mechanisms, triggering conserved pathogen defense pathways in rice [41]. Lesions are also associated with a high level of H_2_0_2_ accumulation. For example, *Spl40* and *OsSPL24* activation of the SA and JA signaling pathways is likely responsible for increased disease resistance [14,18], and the spotted-leaf mutant lm-Zh also showed enhanced resistance to these pathogens, with defense-related genes *OsPR1a*, *OsPR1b, OsPR10*, *OsPAL1*, *OsAOS2*, and *WRKY45* upregulated in lm-Zh leaves [34]. Here, the SA and JA metabolic pathways were activated in the *HM113* mutant, as evidenced by the upregulated expression of genes essential to the JA and SA signaling pathways (Supplemental Data Figure S1). However, the knockout lines showed reduced resistance to *PXO347*, marked by lower gene expression in typical defense responses. The expression levels of these defense response genes returned to wild-type levels, with some even lower than the wild type, indicating a similar response to the pathogen. These results suggested that the mutated gene *HM113* positively regulates the immune response in rice, and the mutation impairs plant growth and development, activates a defense response, and enhances disease resistance. Still, the plant disease resistance mechanism is exceptionally complex.

A single nucleotide mutation, from A to T, was identified at position 847 in the first exon of the gene *LOC_Os07g30510*, which was initially cloned from rice cultivar 93-11 [9]. Although both genes are dominant, we were unable to observe the overexpression phenotype of *HM113* in the wild type, unlike Zhou et al., who achieved the phenotype by overexpressing *LIL1* in a wild-type background. Instead, we obtained the desired result only by knocking out the mutant allele using CRISPR/Cas9. *HM113* and *LIL1* are both lesion-mimic mutants that can help us understand the hypersensitive response (HR), such as cell death, which is controlled in rice; however, their genetic composition and mechanism may vary. *HM113* exhibited a single base substitution (A847T) and displayed a dominant gain-of-function phenotype, whereas *LIL1* has a (Val429 to Ile) mutation in the kinase domain, acting as a semi-dominant allele. When we knocked out the *HM113* gene using CRISPR/Cas9, the phenotype of the knockout lines was similar to that of the wild-type, resulting in the elimination of the mutant phenotype. In contrast, the overexpression of *LIL1* induced a *LIL1*-like lesion phenotype in Nipponbare and also displayed increased expression of defense-related genes, as well as enhanced resistance to the rice blast fungus and enhanced ROS accumulation. Both of these mutants, however, exhibited disease resistance and HR-like cell death. While the mutant *LIL1* exhibited a disruption of CRK activity, *HM113* showed a gain-of-function signaling, indicating that different mutation pathways can lead to similar immune responses.

The subcellular localization of HM113 was found in the cytosol, although a transmembrane domain (301-323aa) was expected, suggesting that HM113 may be involved in signal transduction or metabolic pathways. However, this needs further investigation, as GFP-based localization and transient overexpression can sometimes mislead in identifying protein targets [42,43]. Other strategies include co-localization with organelle markers, biochemical fractionation, the development of a transmembrane domain-deleted HM113 mutant, and the establishment of stable expression in rice. Immunolocalization or immunogold electron microscopy with a specific antibody can also help determine HM113 localization more accurately [44]. The protein kinase has become a crucial component in plants’ signal transduction pathways in response to numerous environmental signals. The predicted cysteine-rich repeat kinase (CRK), encoded by *LOC_Os07g30510*, belongs to a sub-family that is distinguished by one or more extracellular DUF26 domains with a C-X8-C-X2-C motif. The stress-antifungal domain is the name given to the DUF26 domain [9]. CRKs feature an RLK domain, an extracellular domain for receiving signals, a single-pass transmembrane domain, and a conserved intracellular serine/threonine protein kinase domain responsible for signal transduction [45,46]. These dominant lesions mimic mutant genes, known for their gain-of-function characteristics, which are involved in essential processes such as initiating spontaneous cell death and enhancing plant immunity. By improving our understanding of plant defense mechanisms, stress signaling, and programmed cell death, these genes can promote the development of disease-resistant varieties. However, further investigation is necessary to elucidate the molecular pathways and their potential effects on sustainable rice cultivation. Ultimately, our findings indicated that the mutation in *HM113* causes the spotted-leaf phenotype and that *LOC_Os07g30510* is the target gene of the *HM113* dominant mutant, as confirmed by the use of gene editing technology. After altering the upstream mutation site of the *HM113* gene, which caused a frameshift, the mutation phenotype of *HM113* was eliminated, and resistance to bacterial blight was subsequently lost: this indicates that it functions as a gain-of-function gene.

## 4. Materials and Methods

### 4.1. Plant Material and Growth Conditions

This study utilized the WT IR64 and *HM113* mutants from an EMS-induced mutant bank of IR64. The wild-type IR64 and mutant *HM113* were cultivated in the paddy field during the summer. In contrast, using standard water and fertilizer management, transgenic rice knockout lines were grown in a greenhouse at the China National Rice Research Institute (CNRRI) in Fuyang, Hangzhou, China.

### 4.2. Agronomic Traits Evaluation

After attaining full maturity, the main agronomic characteristics, including plant height, panicle length, 1000-grain weight, and seed-setting rate, were determined from three randomly selected plants of IR64, *HM113*, and the T1 knockout lines. The analysis used the mean of three replicates.

### 4.3. CRISPR/CAS9 Vector Construction

The knockout construct using CRISPR/CAS9 was created using a previously published method by [47], and the mutant calli were modified via Agrobacterium tumefaciens-mediated transformation. The procedure provided by [48] was used for all Agrobacterium tumefaciens transformations.

### 4.4. Physiological Parameters Measurement

During the tillering stage, the total chlorophyll of the knockout lines *HM113* and IR64 was extracted from the top two leaves of the plants. Likewise, the activities of ROS scavenging enzymes, such as catalase (CAT), superoxide dismutase (SOD), and peroxidase (POD), as well as enzymatic activities like H_2_O_2_, MDA, and soluble proteins, were evaluated using the corresponding test kit from the (Nanjing Jiancheng Bioengineering Institute, Nanjing, China) following the manufacturer’s instructions. The mean value of three biological replicates was analyzed using Student’s *t*-test, one-way ANOVA, and Duncan’s test.

### 4.5. Disease Evaluation

At the maximum tillering stage, five fully expanded leaves from *HM113*, IR64, and the knockout lines were selected and inoculated with PX037 bacterial strains using the leaf clipping method described by [49]. The *Xanthomonas oryzae pv. oryzae* strain was grown on WF-P medium (20.0 g sucrose, 5.0 g peptone, 0.5 g calcium nitrate, 0.82 g sodium phosphate, 0.2 g ferrous sulfate, 17.0 g agar in 1 L distilled water). The strain was collected and diluted with sterile water for inoculation, after which the OD600 value was adjusted to 1.0. The length of the disease lesion was measured 14 days after inoculation using a transparent plastic ruler. The disease index (%) was calculated following the leaf-clip inoculation method described by [49], as (lesion length ÷ leaf length) × 100, where lesion length is the distance from the cut site to the visible end of the lesion, and leaf length is the total length of the inoculated leaf. The mean value of five independent leaves was used for analysis, and statistical significance was determined using Student’s *t*-test.

### 4.6. Subcellular Localization

For Subcellular localization, the plasmid P1132 was used to create a GFP vector, which was then examined in rice protoplasts of HM113. The whole coding sequence (CDS) of *HM113* was amplified using a specific primer design. After the PCR was amplified, we purified the PCR product using a Gel extraction and clean-up kit. The PCR product was then merged with the PAN580 vector. The GFP signal was detected by viewing using a Zeiss LSM700 confocal microscope (Carl Zeiss, Oberkochen, Germany) 48 h after transformation.

### 4.7. RNA Extraction and Gene Expression Analysis

To assess the expression level, the total RNA was isolated from various tissues, including roots, shoots, stem, leaf, sheath, flag leaf, node, internode, node 1, internode 1, and panicle of wild-type IR64 using the NucleoZOL reagent (MACHEREY-NAGEL, Düren, Germany), according to the manufacturer’s instructions at various developmental stages. The RNA samples were treated with the PrimeScript™ RT Master Mix (TaKaRa, Dalian, China), and 1 µg was used to synthesize first-strand cDNA for reverse transcription-PCR. Quantitative real-time PCR (qRT-PCR) was performed using PowerUp ™ SYBR ™ Green Master Mix (Thermo Fisher Scientific, Waltham, MA, USA) and a Thermal Cycler Dice Real Time System II (TaKaRa, Dalian, China) according to the manufacturer’s instructions. As an internal control, rice ubiquitin (*LOC_Os03g13170*) was used. Three replicates were performed for each test and were analyzed using the 2^−∆∆Ct^ method.

### 4.8. Map-Based Cloning

The mutation was previously mapped to chromosome 7 by [29]. Using SSR markers and fine-mapping with segregating F_2_ populations, the causal locus was confined to a ~308 kb interval on the long arm of chromosome 7, flanked by markers RM21605 and RM418 [29]. For further fine mapping, F_2_ individual plants of the mutant type were obtained from the crosses *HM113*/Moroberekan, *HM113*/Nekken, and *HM113*/CPSLO17. The Gramene website (http://www.gramene.org/ accessed on 23 June 2024) was used to obtain simple sequence repeat (SSR) markers. After comparing the sequences between the japonica cultivar Nipponbare and the indica cultivar 9311 in the public database on the website (https://ensembl.gramene.org/index.html accessed on 23 June 2024), insertion/deletion (InDel) markers were designed using Primer3 input. Sangon Biotech Co., Ltd. (Shanghai, China) synthesized the primers. The detection and PCR reaction were carried out as previously described by [15]. The Supplementary Table S1 lists the primer sequences used for fine mapping.

## 5. Conclusions

Our study demonstrated that the mutant *HM113* was a gain-of-function gene that encoded a cysteine-rich receptor-like kinase (CRK) which disrupted reactive oxygen species (ROS) and led to excessive H_2_O_2_ accumulation, lesion formation, and programmed cell death. The CRISPR/Cas9 knockout of the mutant allele restored wild-type traits, confirming the gain-of-function nature. These results identify *LOC_Os07g30510* as a key regulator of ROS signalling and defense in rice, providing the basis for developing disease-resistant varieties with improved yield stability.

## Data Availability

Data is contained within the article or Supplementary Materials.

## Supplementary Materials

The following supporting information can be downloaded at: https://www.mdpi.com/article/10.3390/plants14223429/s1, Figure S1: Defense-related gene expression levels in HM113 and IR64 involved in the JA and SA pathways; Figure S2: Analysis of cell death and ROS-associated parameters of IR64 and HM113 at the tillering stage; Figure S3: CRISPR/Cas9 sequence data and its target sequence (ACTGTCGGAGATCAACTCAACGG); Table S1: The primers used in the study are listed. Table S2: Comparison of SNPs and indels within the 308 kb candidate region on chromosome 7 for identifying *HM113*-specific mutations based on resequencing data.

## References

[1] Khan M.A., Naeem M., Iqbal M. (2014). Breeding Approaches for Bacterial Leaf Blight Resistance in Rice (*Oryza sativa* L.), Current Status and Future Directions. Eur. J. Plant Pathol..

[2] Fiyaz R.A., Dustakar S., Chaithanya K., Mounika K., Chiranjeevi M.C., Laha G.S., Viraktamath B.C., Rao L.V.S., Sundaram R.M. (2022). Genetic Improvement of Rice for Bacterial Blight Resistance: Present Status and Future Prospects. Rice Sci..

[3] Korinsak S., Darwell C.T., Wanchana S., Praphaisal L., Korinsak S., Thunnom B., Patarapuwadol S., Toojinda T. (2021). Identification of Bacterial Blight Resistance Loci in Rice (*Oryza sativa* L.) against Diverse *Xoo* Thai Strains by Genome-Wide Association Study. Plants.

[4] Liu W., Liu J., Triplett L., Leach J.E., Wang G.L. (2014). Novel Insights into Rice Innate Immunity against Bacterial and Fungal Pathogens. Annu. Rev. Phytopathol..

[5] Zhang Y., Liu Q., Zhang Y., Chen Y., Yu N., Cao Y., Zhan X., Cheng S., Cao L. (2019). LMM24 Encodes Receptor-Like Cytoplasmic Kinase 109, Which Regulates Cell Death and Defense Responses in Rice. Int. J. Mol. Sci..

[6] Wu C., Bordeos A., Madamba M.R.S., Baraoidan M., Ramos M., Wang G.L., Leung H. (2008). Rice Lesion-Mimic Mutants with Enhanced Resistance to Diseases. Mol. Genet. Genom..

[7] Wu J., Yang R., Yang Z., Yao S., Zhao S., Wang Y., Li P., Song X., Jin L., Zhao J. (2020). Lesion mimic mutants: Unveiling cell death and defense mechanisms in plants. Int. J. Mol. Sci..

[8] Liu Q.N., Ning Y.S., Zhang Y.X., Yu N., Zhao C.D., Zhan X.D., Wu W.X., Chen D.B., Wei X.J., Wang G.L. (2017). OsCUL3a Negatively Regulates Cell Death and Immunity by Degrading OsNPR1 in Rice. Plant Cell.

[9] Zhou Q., Zhang Z., Liu T., Gao B., Xiong X. (2017). Identification and Map-Based Cloning of the Light-Induced Lesion Mimic Mutant 1 (LIL1) Gene in Rice. Front. Plant Sci..

[10] Ma J., Yang S., Wang D., Tang K., Feng X.X., Feng X.Z. (2020). Genetic Mapping of a Light-Dependent Lesion Mimic Mutant Reveals the Function of Coproporphyrinogen III Oxidase Homolog in Soybean. Front. Plant Sci..

[11] Fekih R., Tamiru M., Kanzaki H., Abe A., Yoshida K., Kanzaki E., Saitoh H., Takagi H., Natsume S., Undan J.R. (2015). The Rice (*Oryza sativa* L.) Lesion Mimic Resembling, Which Encodes an AAA-Type ATPase, Is Implicated in Defense Response. Mol. Genet. Genom..

[12] Du D., Liu M., Xing Y., Chen X., Zhang Y., Zhu M., Lu X., Zhang Q., Ling Y., Sang X. (2019). Semi-Dominant Mutation in the Cysteine-Rich Receptor-Like Kinase Gene, ALS1, Conducts Constitutive Defence Response in Rice. Plants.

[13] Yin Z., Chen J., Zeng L., Goh M., Leung H., Khush G.S., Wang G.L. (2000). Characterizing Rice Lesion Mimic Mutants and Identifying a Mutant with Broad-Spectrum Resistance to Rice Blast and Bacterial Blight. Mol. Plant Microbe Interact..

[14] Sathe A.P., Su X., Chen Z., Chen T., Wei X., Tang S., Zhang X.B., Wu J.L. (2019). Identification and Characterization of a Spotted-Leaf Mutant spl40 with Enhanced Bacterial Blight Resistance in Rice. Rice.

[15] Feng B.H., Yang Y., Shi Y.F., Shen H.C., Wang H.M., Huang Q.N., Xu X., Lü X.G., Wu J.L. (2013). Characterization and Genetic Analysis of a Novel Rice Spotted-Leaf Mutant HM47 with Broad-Spectrum Resistance to *Xanthomonas oryzae* pv. *oryzae*. J. Integr. Plant Biol..

[16] Shen H.C., Shi Y.F., Feng B.H., Wang H.M., Xu X., Huang Q., Lü X.G., Wu J.L. (2014). Identification and Genetic Analysis of a Novel Rice Spotted-Leaf Mutant with Broad-Spectrum Resistance to *Xanthomonas oryzae* pv. *oryzae*. J. Integr. Agric..

[17] Shang H., Li P., Zhang X., Xu X., Gong J., Yang S., He Y., Wu J.L. (2022). The Gain-of-Function Mutation, OsSpl26, Positively Regulates Plant Immunity in Rice. Int. J. Mol. Sci..

[18] Chen Z., Chen T., Sathe A.P., He Y., Zhang X.B., Wu J.L. (2018). Identification of a Novel Semi-Dominant Spotted-Leaf Mutant with Enhanced Resistance to *Xanthomonas oryzae* pv. oryzae in Rice. Int. J. Mol. Sci..

[19] Lorrain S., Lin B., Auriac M.C., Kroj T., Saindrenan P., Nicole M., Balagué C., Roby D. (2004). VASCULAR ASSOCIATED DEATH1, a Novel GRAM Domain–Containing Protein, Is a Regulator of Cell Death and Defense Responses in Vascular Tissues. Plant Cell.

[20] Chen X., Hao L., Pan J., Zheng X., Jiang G., Jin Y., Gu Z., Qian Q., Zhai W., Ma B. (2012). *SPL5*, a Cell Death and Defense-Related Gene, Encodes a Putative Splicing Factor 3b Subunit 3 (SF3b3) in Rice. Mol. Breed..

[21] Ma J., Wang Y., Ma X., Meng L., Jing R., Wang F., Wang S., Cheng Z., Zhang X., Jiang L. (2019). Disruption of Gene SPL35, Encoding a Novel CUE Domain-Containing Protein, Leads to Cell Death and Enhanced Disease Response in Rice. Plant Biotechnol. J..

[22] Yamanouchi U., Yano M., Lin H., Ashikari M., Yamada K. (2002). A Rice Spotted Leaf Gene, Spl7, Encodes a Heat Stress Transcription Factor Protein. Proc. Natl. Acad. Sci. USA.

[23] Zeng L.R., Qu S., Bordeos A., Yang C., Baraoidan M., Yan H., Xie Q., Nahm B.H., Leung H., Wang G.L. (2004). Spotted Leaf11, a Negative Regulator of Plant Cell Death and Defense, Encodes a U-Box/Armadillo Repeat Protein Endowed with E3 Ubiquitin Ligase Activity. Plant Cell.

[24] Quezada E.H., García G.X., Arthikala M.K., Melappa G., Lara M., Nanjareddy K. (2019). Cysteine-Rich Receptor-Like Kinase Gene Family Identification in the Phaseolus Genome and Comparative Analysis of Their Expression Profiles Specific to Mycorrhizal and Rhizobial Symbiosis. Genes.

[25] Andaya C.B., Ronald P.C. (2003). A Catalytically Impaired Mutant of the Rice Xa21 Receptor Kinase Confers Partial Resistance to *Xanthomonas oryzae* pv. *oryzae*. Physiol. Mol. Plant Pathol..

[26] Rao Y., Jiao R., Wang S., Wu X., Ye H., Pan C., Li S., Xu D., Zhou W., Dai G. (2021). SPL36 Encodes a Receptor-Like Protein Kinase That Regulates Programmed Cell Death and Defense Responses in Rice. Rice.

[27] Hong S.W., Jon J.H., Kwak J.M., Nam H.G. (1997). Identification of a Receptor-Like Protein Kinase Gene Rapidly Induced by Abscisic Acid, Dehydration, High Salt, and Cold Treatment in Arabidopsis thaliana. Plant Physiol..

[28] Zhao Y., Wu G., Shi H., Tang D. (2019). Receptor-Like Kinase 902 Associates with and Phosphorylates Brassinosteroid-Signaling Kinase 1 to Regulate Plant Immunity. Mol. Plant.

[29] Guo D., Shi Y.F., Wang H.M., Zhang X.B., Song L.X., Xu X., He Y., Guo L., Wu J.L. (2016). Characterization and Gene Fine Mapping of a Rice Dominant *Spotted-Leaf* Mutant. Acta Agron. Sin..

[30] Kang S.G., Lee K.E., Singh M., Kumar P., Matin M.N. (2021). Rice Lesion Mimic Mutants (*Lmm*): The Current Understanding of Genetic Mutations in the Failure of ROS Scavenging during Lesion Formation. Plants.

[31] Qi P., Tian M., Yang S., Shui Y., Li P., Yin W., Li Q., Bai D., Huang Q., Li Y. (2024). Phenotypic Characterization and Gene Mapping of the Lesion Mimic Mutant *lmm28* in Rice. Agronomy.

[32] Chen T., Chen Z., Sathe A.P., Zhang Z., Li L., Shang H., Tang S., Zhang X., Wu J. (2019). Characterization of a Novel Gain-of-Function *Spotted-Leaf* Mutant with Enhanced Disease Resistance in Rice. Rice Sci..

[33] Li P., Shang H., Xu X., Gong J., Wu J.L., Zhang X. (2024). A Novel Single Base Mutation in *OsSPL42* Leads to the Formation of Leaf Lesions in Rice. Int. J. Mol. Sci..

[34] Sun L., Wang Y., Liu L.L., Wang C., Gan T., Zhang Z., Wang Y., Wang D., Niu M., Long W. (2017). Isolation and Characterization of a *Spotted Leaf 32* Mutant with Early Leaf Senescence and Enhanced Defense Response in Rice. Sci. Rep..

[35] Asada K. (2006). Production and Scavenging of Reactive Oxygen Species in Chloroplasts and Their Functions. Plant Physiol..

[36] Kawano T. (2003). Roles of the Reactive Oxygen Species-Generating Peroxidase Reactions in Plant Defense and Growth Induction. Plant Cell Rep..

[37] Whitaker C., Beckett R.P., Minibayeva F.V., Kranner I. (2010). Production of Reactive Oxygen Species in Excised, Desiccated and Cryopreserved Explants of *Trichilia dregeana* Sond. S. Afr. J. Bot..

[38] Bansal R., Rana N., Singh A., Dhiman P., Mandlik R., Sonah H., Deshmukh R., Sharma T.R. (2020). Evolutionary Understanding of Metacaspase Genes in Cultivated and Wild *Oryza* Species and Its Role in Disease Resistance Mechanism in Rice. Genes.

[39] Apel K., Hirt H. (2004). Reactive Oxygen Species: Metabolism, Oxidative Stress, and Signal Transduction. Annu. Rev. Plant Biol..

[40] Matin M.N., Pandeya D., Baek K.-H., Lee D.-S., Lee J.-H., Kang H.-D., Kang S.-G. (2010). Phenotypic and Genotypic Analysis of Rice Lesion Mimic Mutants. Plant Pathol. J..

[41] Sharma A., Gupta R., Mehta P. (2023). Regulation of Plant Defense Mechanisms by Salicylic Acid and Jasmonic Acid in Rice. Plant Sci. J..

[42] Snapp E.L. (2005). Design and use of fluorescent fusion proteins in cell biology. Curr. Protoc. Cell Biol..

[43] Bhat R.A., Lahaye T., Panstruga R. (2006). The visible touch: In planta visualization of protein–protein interactions by fluorophore-based methods. Plant Methods.

[44] Von Heijne G. (2006). Membrane-protein topology. Nat. Rev. Mol. Cell Biol..

[45] Chen Z. (2001). A Superfamily of Proteins with Novel Cysteine-Rich Repeats. Plant Physiol..

[46] Chern M., Xu Q., Bart R.S., Bai W., Ruan D., Sze-To W.H., Canlas P.E., Jain R., Chen X., Ronald P.C. (2016). A Genetic Screen Identifies a Requirement for Cysteine-Rich Receptor-Like Kinases in Rice NH1 (*OsNPR1*)-Mediated Immunity. PLoS Genet..

[47] Ma X., Zhang Q., Zhu Q., Liu W., Chen Y., Qiu R., Wang B., Yang Z., Li H., Lin Y. (2015). A Robust CRISPR/Cas9 System for Convenient, High-Efficiency Multiplex Genome Editing in Monocot and Dicot Plants. Mol. Plant.

[48] Toki S., Hara N., Ono K., Onodera H., Tagiri A., Oka S., Tanaka H. (2006). Early Infection of Scutellum Tissue with *Agrobacterium* Allows High-Speed Transformation of Rice. Plant J..

[49] Kauffman H.E. (1973). An Improved Technique for Evaluating Resistance of Rice Varieties to *Xanthomonas oryzae*. Plant Dis. Rep..

