# Supplementary material for: Functional Characterization of Rice Spotted-Leaf Mutant HM113 Reveals an Amino Acid Substitution in a Cysteine-Rich Receptor-like Kinase
Source: Plants (Basel). 2025 Nov 9;14(22):3429. doi: 10.3390/plants14223429 (PMC12655428; doi:10.3390/plants14223429)
Supplement: Supplementary file 1 [file plants-14-03429-s001.zip › Supplementary .pdf]

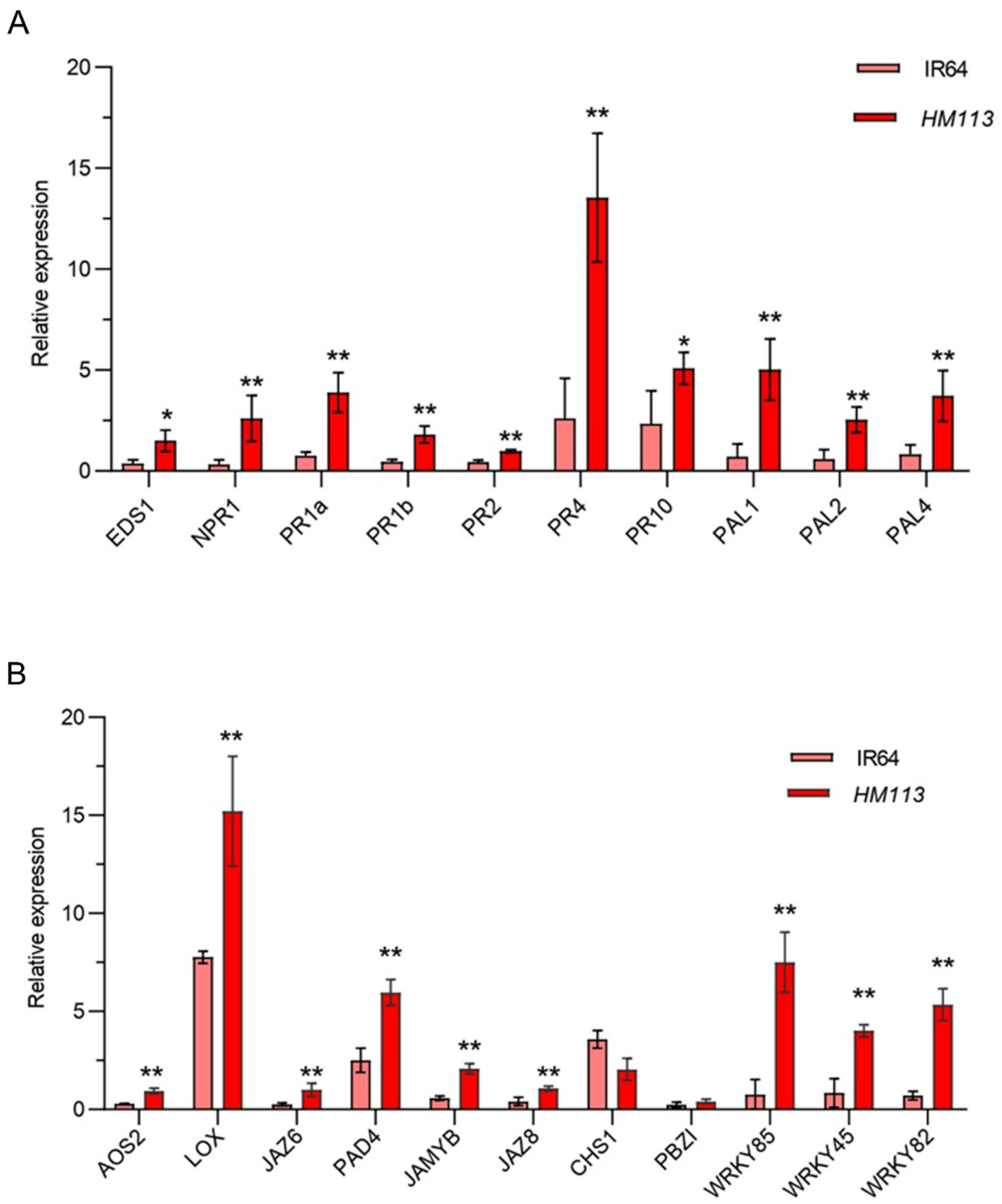

Figure S1: Defense-related gene expression levels in *HM113* and IR64 involved in the JA and SA pathways

(A) JA signaling pathway genes analysis **(B).** SA signaling pathway genes analysis. By student's t-test, ** means significance at P ≤0.01, and * indicates significance at P ≤0.05. Values are means ± SD (n=3).

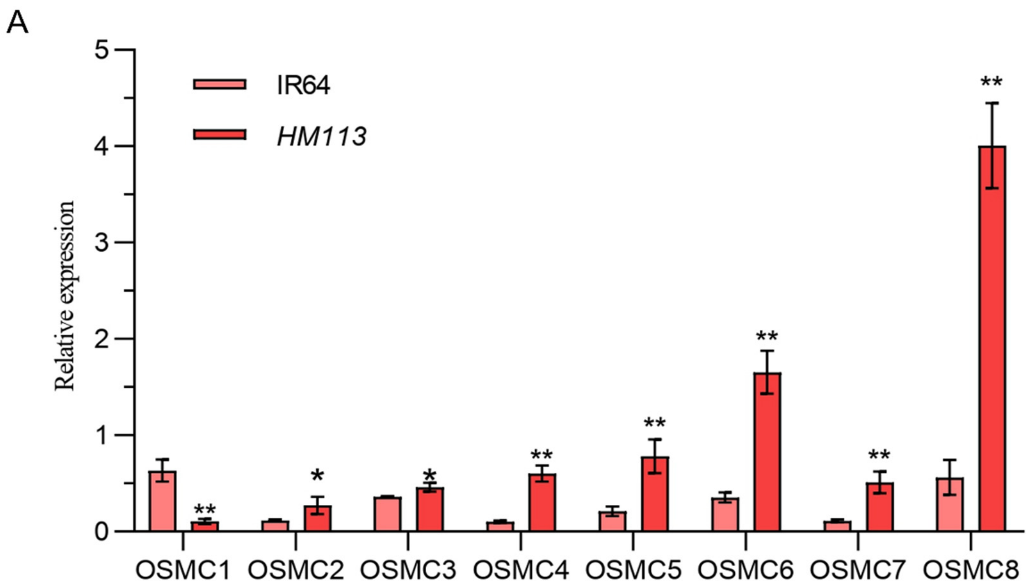

Figure S2: Analysis of cell death and ROS-associated parameters of IR64 and *HM113* at the tillering stage.

Relative expression of *Oryza sativa* metacaspase genes OsMC1, OsMC2, OsMC2, OsMC3, OsMC4, OsMC5, OsMC6, and OsMC7. Student's t-test, ** means significance at P ≤0.01, and * indicates significance at P ≤0.05. Values are means ± SD (n=3).

CR-1 (-2bp):

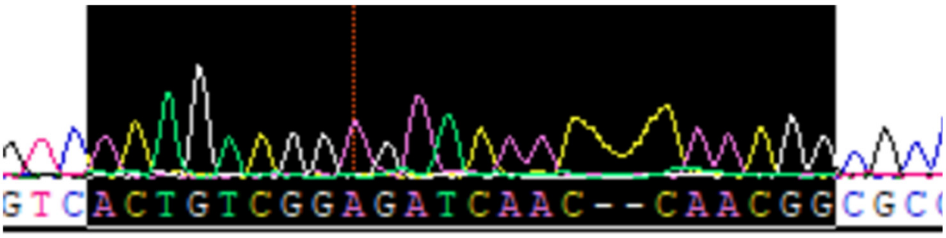

CR-6 (+1bp):

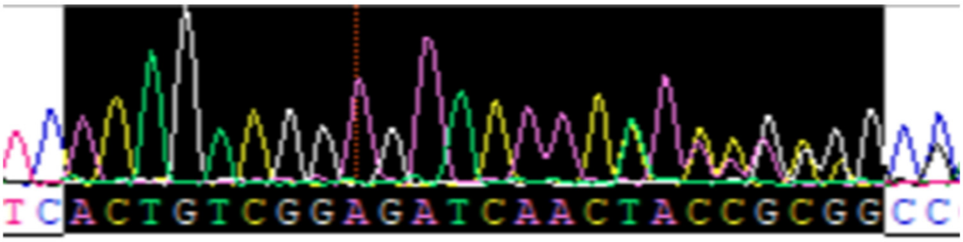

CR-9 (-2bp):

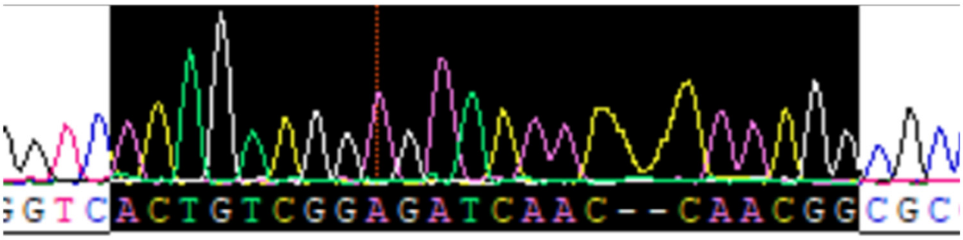

Figure S3: CRISPR/Cas9 sequence data and its target sequence *(*ACTGTCGGAGATCAACTCAACGG*)*.

Table S1: The primers used in the study are listed.

Name	Primer sequence (5'→3')	Purpose
OsMC1-F	GCTTCATCAAGGCGGTGGAGTG	
OsMC1-R	AAGTTGGCGACCTTGCGGATG	
OsMC2-F	CGACCCGTACAGGGTGCCGA	
OsMC2-R	GCACAGCGCCTCGTCGTAGC	
OsMC3-F	GGCTCCTTCGTCCGCAAGAT	
OsMC3-R	CACAGGAGAAACGGTTTCCTGT	
OsMC4-F	TCGACGTTCGTGGAGATGCTC	
OsMC4-R	ATTCACGAGCCGCCTGATCTT	PCD related genes
OsMC5-F	GTGCCAGACCGACCAGACAT	
OsMC5-R	CCGCTCTTCTCCGACAGGAT	
OsMC6-F	CCACACCGCAGGGTTCTTCAT	
OsMC6-R	GTCCAGGCTGCTGAGTGTATCC	
OsMC7-F	ATACAGACCGTGCTGGCGTC	
OsMC7-R	AGGAATGGCGTCTCGGCGTT	
OsMC8-F	TCCGGCAAGTGCCTCGTAAC	
OsMC8-R	CAATGCGGTCGGTCACAGGAT	
AOS2-F	CTCGTCGGAAGGCTGTTGCT	
AOS2-R	ACGATTGACGGCGGAGGTT	
LOX-F	GATGGCGGTGCTCGACGTGCT	
LOX-R	GCACCTGTTCTTGAGCTTTCTAT	
JAZ6-F	GGACATGCCGATCGCGAGGAA	
JAZ6-R	GCGCGAGTGCATGTGTCCAA	
PAD4-F	CCAACATGTACCGCATCAAG	
PAD4-R	GGTTGTTTCGGTGGTAGTGGC	PR genes
JAMyb-F	CCGAGCATGGTGACTAGCTCATCTT	
JAMyb-R	CCTTGCACCCAACCGTTAAGCTGTT	
JAZ8-F	CCAAACACGGCGGAAACAG	
JAZ8-R	GGTGGACGGGAAGTTCTCAAAG	
CHS1-F	GACTACCCGGACTACTACTTCA	
CHS1-R	CTTCCTGATCTGCGACTTG	
PBZ1-F	CCCTGCCGAATACGCCTAA	

PBZ1-R	CTCAAACGCCACGAGAATTTG
WRKY85-F	CAGCAAGAAAAGGAATATACAAAT
WRKY85-R	CTCAATGTGTTTCCTAACATTACA
WRKY45-F	TTCCTTGTTGATGTGTCGTCTCA
WRKY45-R	CCCCCAGCTCATAATCAAGAAC
WRKY82-F	AGTGAAAAGTAGTGAAAATTCCAG
WRKY82-R	GTGCTAGTTTCAATTATTCTGCTTCGT
PR1a-F	GGAAGTACGGCGAGAACATC
PR1a-R	TGGTCGTACCACTGCTTCTC
PR1b-F	AGAACTACGCCAGCCAGAGAAG
PR1b-R	TTCTCGCCAAGGTTGTTCCG
PR2-F	GGCAGGTGAGAGTCTACGAGGAA
PR2-R	GCTGTCATCCGAGCTAAGTGTT
PR3-F	CGTGTCTGTGGAGAGCGTGGTC
PR3-R	TCGTCGTTGGTGCGGTCATTGG
PR4-F	AGTATGGATGGACCGCCTTCTGT
PR4-R	CTCGCAATTATTGTCGCACCTGTTC
NPR1-F	GGCAGGTGAGAGTCTACGAGGAA
NPR1-R	GCTGTCATCCGAGCTAAGTGTT
PAL1-F	TCGGCTGCGTATTCCTCA
PAL1-R	AGTTGATGGGAAGGGGCT
PAL2-F	GCATCAGCTTCCAACTCG
PAL2-R	GGTTTCGCACTCCATTACAGA
PAL4-F	CTTCACAACAGCTAATCGAG
PAL4-R	CGCACTCCATTTCAGTACCA
EDS1-F	CATTCCAAGAACGAGGACACTG
EDS1-R	CAAGACTCAAGGCTAGAACCGA

RM418-F	TCGCGTATCGTCATGCATA	
RM418-R	GAGCACATATGCCACGTACG	
RM21605-F	GACGCCTCCTTGAGTATTTCACC	
RM21605-R	CTCCGTGCTCATTGTGAGAAACC	Fine Mapping
RM1135-F	AGCCAACCAAGCAAGATAGC	
RM1135-R	ACACACATGTAAGCCTCCCC	

RM432-F	TTCTGTCTCACGCTGGATTG	
RM432-F	AGCTGCGTACGTGATGAATG	
QPCR-F	GGTGGGCACTTATGGGTATATG	
QPCR-R	AGAACCAGAACTCCAAAGCTAAA	qRT-PCR
Ubi-F	CCCTCCACCTCGTCCTCAG	
Ubi-R	AGATAACAACGGAAGCATAAAAGTC	Reference gene
113-F	TGAGGCTCTACGACGTCGATCGC	Construction
113-R	CCTATGCCTTGGCACGGTGGG	
